# Validation of the SQUASH physical activity questionnaire using accelerometry: The NEO study

**DOI:** 10.1016/j.ocarto.2024.100462

**Published:** 2024-03-18

**Authors:** Sietse E.S. Terpstra, Lotje A. Hoogervorst, Jeroen H.P.M. van der Velde, Renée de Mutsert, Lotte A. van de Stadt, Frits R. Rosendaal, Margreet Kloppenburg

**Affiliations:** a Department of Rheumatology, Leiden University Medical Center, the Netherlands; b Department of Clinical Epidemiology, Leiden University Medical Center, the Netherlands; c Department of Orthopaedics, Leiden University Medical Center, the Netherlands; d Department of Medical Decision Making, Leiden University Medical Center, Leiden, the Netherlands

**Keywords:** Physical activity, Patient reported outcomes, Quality of life, Epidemiology, Accelerometer

## Abstract

**Objective:**

To investigate the construct validity of the SQUASH (Short QUestionnaire to ASsess Health-enhancing physical activity).

**Design:**

This is a cross-sectional analysis using baseline measurements from middle-aged participants in the Netherlands Epidemiology of Obesity (NEO) study. The SQUASH consists of questions on eleven physical activities investigating days per week, average duration per day and intensity, leading to a summed score in Metabolic Equivalent of Task hours (MET h) per week. To assess convergent validity, a Spearman's rank correlation between SQUASH and ActiHeart was calculated. To assess extreme group validity, three groups expected to differ in SQUASH total physical activity outcome were compared. For discriminative validity, a Spearman's rank correlation between SQUASH physical activity and participant height was investigated.

**Results:**

SQUASH data were available for 6550 participants (mean age 56 years, 44% men, mean BMI 26.3, 15% with knee OA, 13% with hand OA). Median physical activity (interquartile range) was 118 (76; 154) MET h/week according to SQUASH and 75 (58; 99) according to ActiHeart. Convergent validity was weak (rho ​= ​0.20). For all three extreme group comparisons, a statistically significant difference was present. Discriminative validity was present (rho ​= ​0.01). Compared with the reference quintile, those with a discrepancy SQUASH ​> ​ActiHeart and SQUASH ​< ​ActiHeart were relatively younger and more often male.

**Conclusions:**

The construct validity of the SQUASH seems sub-optimal. Physical activity reported by the SQUASH was generally higher than reported by ActiHeart. Whether the differences between SQUASH and ActiHeart are e.g. due to different underlying domains, limitations to our study, or reflect true differences needs further investigation.

## 1 Introduction

Adequate physical activity levels have been associated with wide-ranging beneficial health outcomes, such as a lowered risk of cardiovascular disease, type 2 diabetes, hypertension, osteoarthritis, cancer, and all-cause mortality [1,2]. Therefore, there are several guidelines for physical activity, provided for example by governments of the United States, United Kingdom, and the Netherlands [[3], [4], [5]]. These guidelines recommend a minimal amount of physical activity per week. Consequently, for public health research it is important to adequately measure physical activity.

Instruments to measure physical activity can broadly be divided into two methods: subjective (e.g., interviews and questionnaires) and objective (e.g., accelerometers, pedometers, and activity observation) [6]. Both these methods have advantages and disadvantages. Self-reported measurements can provide detail or context on which specific domains of physical activity are performed (e.g., cycling, doing groceries), while most objective methods cannot distinguish this well [7]. In addition, self-reported measurements can be faster and more cost-effective than objective methods. However, objective methods have the advantage of being the result of wearing a device and are therefore not dependent on the memory of the user [6,7], whereas self-reported methods are prone to response biases such as recall bias and social desirability bias [8]. Objective methods might be burdensome for users, as these often require carrying an instrument. This burden might cause individuals to abstain from wearing the instrument during some or all activities, leading to underestimation of physical activity [7].

It is important to measure physical activity with instruments with good metric properties. A questionnaire often used is the “SQUASH” (Short QUestionnaire to ASsess Health-enhancing physical activity) [3,9]. The SQUASH is a feasible instrument due to its shortness and has shown test-retest reliability [9,10]. Validity of the SQUASH is less well-established. Validity of instruments is often determined using a “golden standard”. However, as existence of this golden standard is debated for physical activity, information on construct validity of the SQUASH is imperative, which is defined as “the ability of the test to actually measure the intended construct” [11]. Important aspects of construct validity are convergent validity, discriminative validity and extreme group validity [12]. Convergent validity is present if the instrument under investigation correlates with another instrument with which it should correlate (e.g. another physical activity instrument). Extreme group validity is whether extreme groups have different outcomes according to the instrument under investigation. Discriminative validity is present if it does not correlate with an instrument with which it should not correlate. Studies assessing construct validity of the SQUASH showed extreme group validity as well as convergent validity [9,10,13]. Validity of the SQUASH seems to vary between groups with different participant characteristics such as sex and age [[13], [14], [15]], and most studies on validity of the SQUASH are small. Data on validity of the SQUASH are lacking for middle-aged individuals from the general population, despite inactivity-related diseases being highly prevalent in this category of age [16]. It has been shown that physical activity energy expenditure outcomes are higher using the SQUASH than with another questionnaire (“OBiN”) [17]. At the same time, studies that compare the SQUASH with specifically the ActiHeart accelerometer were not found.

Consequently, further validation of the SQUASH by comparison with an objective measure is important. Therefore, we aimed to assess the construct validity of the SQUASH in a general middle-aged population. Furthermore, we investigated participant characteristics associated with difference in outcome between the SQUASH and the ActiHeart.

## 2 Materials and methods

### 2.1 Study design and population

In the NEO study, individuals with a BMI of 27 ​kg/m^2^ or higher were oversampled. To correctly represent baseline associations in the general population, adjustments for the oversampling of individuals with a BMI >27 ​kg/m^2^ were made. This was done by weighting all participants toward the BMI distribution of participants from the Leiderdorp municipality [18], whose BMI distribution was similar to the BMI distribution of the general Dutch population [19]. All results were based on weighted analyses. Consequently, the results apply to a population-based study without over-sampling of individuals with a BMI >27 ​kg/m^2^. We performed a cross-sectional analysis of baseline measurements. Participants with >0 queries missing in the SQUASH were excluded and participants with a valid ActiHeart time of <24 ​hrs were excluded from the analyses involving the ActiHeart. Data collection started in September 2008 and was completed at the end of September 2012. The Medical Ethical Committee of the Leiden University Medical Center (LUMC) approved the design of the study. All participants gave their written informed consent.

### 2.2 The SQUASH questionnaire

The SQUASH includes eleven questions on activities within the domains of leisure time, commuting, work, and household physical activity in an average week in the last month [9]. Each question consists of three queries: days per week, average duration per day, and intensity of activity. Intensity was divided into “light” (<3.0 Metabolic Equivalents of Task (METs)/hour), “moderate” (3.0–6.0 METs/hour) and “vigorous” (>6.0 METs/hour). Items were converted to METs, derived from Ainsworths's compendium of physical activity [20], and to hours/week based on reported frequency and duration of the activities, as described by Wendel-Vos [9]. SQUASH items were combined to calculate a total physical activity level in MET hours/week.

### 2.3 Accelerometer

In order to assess convergent validity, physical activity was also measured for the duration of four consecutive prospective days by an accelerometer in a random subset (14%) of the study population. An accelerometer was combined with two electrocardiogram electrodes (ActiHeart, CamNtech Ltd, United Kingdom), which was placed on the chest of the participants at the level of the third intercostal space. This combined heart rate monitor and accelerometer simultaneously measures heart rate and uniaxial (vertical when standing up) acceleration of the torso. Using a branched equation algorithm, the acceleration and heart rate information was translated into calibrated estimates of physical activity energy expenditure [21,22], which has been shown to have technical reliability and validity [21,23]. In order to allow comparison with the SQUASH, we extrapolated outcomes of the ActiHeart from four days to one week by dividing by four and multiplying by seven. Also, we converted the data from the ActiHeart (kJ/kg/day) to MET-hours per week by dividing by 4.2 (conversion factor from kJ to MET) and subsequently multiplying by 7 (conversion factor from day to week) [24].

### 2.4 Other questionnaires and physical examination

Participants completed a general questionnaire to report demographic, lifestyle, and clinical information. Health-related quality of life was measured with the Short Form (SF)-36 [25]. The physical health summary component score (PCS) and mental health summary component score (MCS) were calculated. Age- and sex-specific Dutch population-based norm scores were used to derive norm-based scores with a mean of 50 and a standard deviation (SD) of 10 [26]. Higher SF-36 scores represent better quality of life.

Trained research nurses measured weight and height in order to calculate BMI (kg/m^2^). In addition, extensive physical examination of the hands and knees was performed by the same research nurses using a standardized scoring form, in order to determine presence of hand and knee osteoarthritis according to the clinical criteria of the American College of Rheumatology [27,28].

### 2.5 Statistical analyses

For individuals from Leiden and its surroundings (n ​= ​4541), oversampling was done of individuals with BMI ≥27 ​kg/m^2^. In order to correctly represent associations in the general Dutch population, individuals from Leiderdorp without any oversampling were included (n ​= ​1671), as the BMI distribution of this municipality is representative for the general Dutch population [19]. By weighting the BMI of our study to the general Leiderdorp population, our results are representative to the general Dutch population.

Convergent validity was assessed by calculating Spearman's rank correlation coefficient between the SQUASH and ActiHeart. Correlation coefficients were interpreted as: (−)0.9 to (−)1.0: very strong; (−)0.7 to (−)0.9: strong; (−)0.4 to (−)0.7: moderate; (−)0.1 to (−)0.4: weak; (−)0.0 to (−)0.1; negligible [29]. Convergent validity was regarded as “present” in case of a positive correlation with ActiHeart and at least “moderate” strength. Extreme group validity was regarded “present” when SQUASH outcomes differed between the extreme groups of: 1) “number of comorbidities” (extreme groups: zero comorbidities versus more than one) 2) “physical activity according to ActiHeart” (lowest versus highest quartile of participants) and 3) “BMI” (participants <25 ​kg/m^2^ versus >30 ​kg/m^2^), using linear regression models. Discriminative validity was assessed by calculating whether the correlation between participant's height and SQUASH total activity was “weak” or less.

Participant characteristics possibly associated with differences between the SQUASH and ActiHeart outcome were analysed by subtracting ActiHeart activity from SQUASH activity, where we grouped participants into quintiles of this difference. As a reference group, the quintile with the smallest difference between the SQUASH and ActiHeart physical activity was chosen, as this allowed for comparisons with groups that had lower or higher outcomes on the SQUASH than with ActiHeart. The following participant characteristics were investigated: age, sex, BMI, ethnicity (white versus non-white), educational level (high versus low), any comorbidity, knee osteoarthritis, hand osteoarthritis, SF-36 PCS and SF-36 MCS. For all participant characteristics, a Jonckheere-Terpstra test was performed between all quintiles. In case the Jonckheere-Terpstra test indicated a difference between the groups (p ​< ​0.05), outcomes of all quintiles for the concerning participant characteristic were compared with the reference quintile. This was done by calculating odds ratios (ORs) using logistic regression in case of dichotomous outcomes and calculating mean difference using linear regression for continuous outcomes, adjusted for age and sex. R Studio Version April 1, 1717 and SPSS version 23.0 (IBM, Armonk, NY, USA) were used for the analyses.

## 3 Results

### 3.1 Study population

The NEO study population consists of 6671 participants. As 121 participants did not fill in the SQUASH completely, the study population for the present analyses consisted of 6550 participants. Of the random subset receiving ActiHeart (955 participants), 62 were excluded due to valid wear time <24 ​h and 18 due to incomplete SQUASH data. Therefore, 875 participants were included in the analyses involving ActiHeart. The number and percentage missing of all included variables can be found in Supplementary Table S1, and did not exceed 1% for any variable. Participant characteristics of the population are shown in Table 1.

**Table 1:** Participant characteristics of the study population.

	Total study group
**General participant characteristics**	
Age (years) (mean, SD)	56 (6)
Sex (male)	44
BMI (kg/m^2^) (mean, SD)	26.3 (4.4)
Overweight (BMI <25)	42
Obesity (BMI >30)	16
Ethnicity (white)	95
Education (high)	46
Any comorbidity	24
More than one comorbidity	4
Knee OA, yesˆ	15
Hand OA, yesˆ	13
SF-36 PCS (mean, SD)	53 (9)
SF-36 MCS (mean, SD)	51 (9)
**Physical activity outcomes**	
SQUASH physical activity (MET h/week)†	118 (76; 154)
Light activity (0–3 METs/hour, MET h/week)†	53 (20; 93)
Moderate activity (3–6 METs/hour, MET h/week)†	42 (22; 74)
Vigorous activity (>6 METs/hour, MET h/week)†	0 (0; 7)
ActiHeart activity (MET h/week)†	72 (55; 94)
SQUASH minus ActiHeart activity (MET h/week) (mean, SD)	47 (63)

Results are based on analyses weighted towards the BMI distribution of the general population. Numbers represent percentage unless specified otherwise. SF-36 scores are norm based with mean of 50, higher scores are better. Abbreviations: SQUASH=Short Questionnaire to Assess Health-Enhancing physical activity. BMI ​= ​body mass index (kg/m^2^), n ​= ​number, OA ​= ​osteoarthritis, SF ​= ​short form, PCS=Physical Component Scale, MCS ​= ​Mental Component Scale, MET ​= ​Metabolic Equivalent of Task. ˆ ​= ​Osteoarthritis according to the clinical criteria of the American College of Rheumatologists. † ​= ​median (Interquartile Range).

**Fig. 1:**
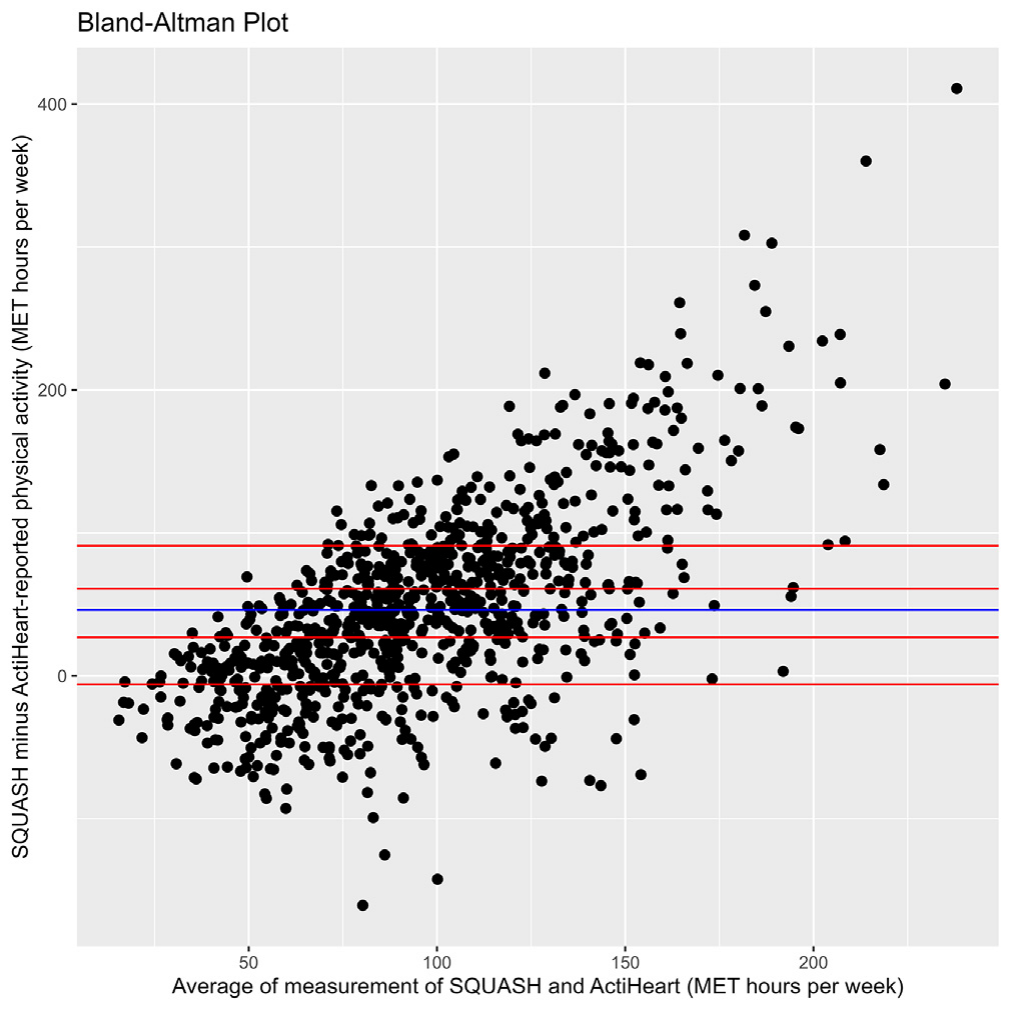
Bland-Altman plot of SQUASH and ActiHeart physical activity. The blue line signifies the mean difference between these two instruments and the red lines signify quintiles of participants in terms of difference between SQUASH and Actiheart.

According to the SQUASH, median (interquartile range (IQR)) total physical activity was 118 (76; 154) MET hours/week of which a median of 45 (IQR 0; 87) MET hours/week was spent on work, 30 (16; 50) on leisure time, 20 (7; 39) on household activities, and 0 (0; 9) on commuting. According to the ActiHeart, a median of 75 (58; 99) MET hours/week of total physical activity was performed. Mean difference between the SQUASH and ActiHeart was 47 (SD 63) MET hours/week, which is equivalent to 5 ​h of vigorous sports per week. The Bland-Altman plot of the difference between the SQUASH and ActiHeart measurement per participant is shown in Fig. 1.

### 3.2 Construct validity

The convergent validity (Spearman rank correlation) was weak (rho ​= ​0.20, p ​< ​0.01, rho ​= ​0.21 for men and rho ​= ​0.20 for women). Extreme group validity was present; participants with any comorbidity had −13 (95% confidence interval (95%CI) −17;-9) MET hours/week according to SQUASH compared with those without any comorbidity, participants with the 25% highest ActiHeart outcome had 38 MET hours/week more SQUASH activity ((95%CI 27; 50) than participants with the 25% lowest ActiHeart outcome, and participants with BMI >30 ​kg/m^2^ had 10 (14; 7) MET hours/week less compared with BMI <25 ​kg/m^2^. Discriminative validity was also present, as a “negligible” Spearman rank correlation between height and SQUASH physical activity was present (rho ​= ​0.01, p ​= ​0.41).

### 3.3 Participant characteristics associated with difference between SQUASH and ActiHeart

For reference, the second quintile was chosen as difference between “SQUASH minus ActiHeart activity” was smallest: was −6 to 27 MET hours/week. The highest (fifth) quintile of participants had 91 to 411 MET hours/week of SQUASH minus ActiHeart activity (Table 2). The lowest quintile had a range of SQUASH minus ActiHeart activity of −161 to −6 MET hours/week.

**Table 2:** Participant characteristics of each quintile of participants concerning SQUASH minus ActiHeart physical activity, and differences with the reference quintile in these characteristics. Q1 is the lowest quintile of SQUASH minus ActiHeart activity and Q5 the highest.

Total group with ActiHeart (14% of study population)	Q1	Q2 (reference quintile)	Q3	Q4	Q5
SQUASH minus ActiHeart (MET h/week, range)	−161;-6	−6; 27	27; 61	61; 91	91; 411
Age	56.5 (6.2)	57.4 (6.0)	55.4 (6.1)	55.7 (5.7)	55.3 (6.0)
*Difference∗*	*−0.9 (−2.2;0.3)*	*-*	*−2.0 (−3.2;-0.7)*	*−1.8 (−3.0;-0.5)*	*−2.1 (−3.4;-0.9)*
Sex (male) ^§^	89 (50%)	54 (30%)	61 (33%)	78 (44%)	91 (50%)
*OR*†	*2.35 (1.52;3.64)*	*-*	*1.19 (0.76;1.87)*	*1.89 (1.23;2.94)*	*2.47 (1.61;3.84)*
BMI	26.8 (3.8)	26.0 (4.0)	26.4 (4.5)	25.7 (4.5)	26.3 (4.6)
*Difference∗*	*0.7 (−0.1;1;6)*	*-*	*0.4 (−0.5;1.3)*	*−0.4 (−1.3;0.4)*	*0.3 (−0.6;1.2)*
Comorbidity^§^	46 (27%)	52 (30%)	30 (17%)	50 (29%)	35 (20%)
*OR*†	*0.87 (0.55;1.39)*	*-*	*0.49 (0.29;0.82)*	*0.95 (0.60;1.51)*	*0.59 (0.36;0.97)*
Knee OA, yes^§^	38 (21%)	28 (15%)	14 (8%)	24 (13%)	25 (13%)
*OR*†	*1.47 (0.86;2.54)*	*-*	*0.47 (0.24;0.93)*	*0.87 (0.48;1.56)*	*0.89 (0.49;1.59)*
Hand OA, yes^§^	11 (5%)	27 (15%)	13 (8%)	27 (15%)	29 (16%)
*OR*†	*0.35 (0.16;0.72)*	*-*	*0.45 (0.22;0.88)*	*0.99 (0.55;1.76)*	*1.05 (0.59;1.86)*
SF-36 PCS	54 (8)	53 (10)	54 (7)	54 (8)	55 (8)
*Difference∗*	*1 (−1;3)*	*-*	*1 (−1;3)*	*1 (−1;3)*	*2 (1;4)*

Results are based on analyses weighted towards the BMI distribution of the general population. Numbers represent mean (standard deviation) unless specified otherwise. SF-36 scores are norm based with mean of 50, higher scores are better. Abbreviations: h ​= ​hour, MET ​= ​Metabolic Equivalent of Task, OR ​= ​odds ratio, CI ​= ​confidence interval, SQUASH=Short Questionnaire to Assess Health-Enhancing physical activity. BMI ​= ​body mass index, OA ​= ​osteoarthritis, SF ​= ​short form, PCS=Physical Component Scale, ˆ ​= ​Osteoarthritis according to the clinical criteria of the American College of Rheumatologists. ∗ ​= ​mean difference compared with reference quintile (quintile 2), with 95% confidence interval, § ​= ​number (percentage), † ​= ​odds ratio for the concerning outcome compared with the reference quintile, with 95% confidence interval.

For seven participant characteristics, there was a difference between any of the quintiles (Jonckheere-Terpstra test <0.05), these characteristics were: age, sex, BMI, any comorbidity, knee osteoarthritis, hand osteoarthritis and SF-36 PCS. When comparing individual quintiles with the reference quintile, we observed differences (significance level <0.05) between the reference quintile and all other quintiles concerning age and sex (Table 2). Compared with the reference quintile, mean age was lower in all quintiles. Men were more represented in the lowest quintile of SQUASH minus ActiHeart activity and in the higher quintiles. For any comorbidity, differences between the third and fifth quintile versus the reference quintile were found. For knee osteoarthritis, a difference between the third and the reference quintile was present. For hand osteoarthritis, a difference was found between both the first and third quintile versus the reference quintile, and for SF-36 PCS this was found for the fifth quintile versus the reference quintile. For BMI, there were no difference between the reference quintile and any other quintile. No dose-response relationships were seen between participant characteristics and amount of over- or underestimating physical activity on the SQUASH compared with the ActiHeart, except for sex (odds ratio).

After adjustment for sex, mean age remained lower in all quintiles than in the reference quintile. After adjustment for age, men remained relatively more represented in the first, fourth and fifth quintile versus the reference quintile. For the other participant characteristics we investigated, the aforementioned differences in participant characteristics with the reference quintile became smaller when adjusting for age and sex. The OR for having any comorbidity for quintile three versus the reference quintile became smaller, as well as the OR for hand osteoarthritis for quintile one versus the reference quintile. The other differences in participant characteristics of any quintile with the reference quintile were no longer statistically significant after adjustment for age and sex, as the effect sizes decreased (Supplement 1).

## 4 Discussion

We investigated the construct validity of the SQUASH questionnaire in the general Dutch middle-aged population, as well as possible determinants of differences between SQUASH and ActiHeart accelerometer outcome in total physical activity energy expenditure.

Convergent validity was absent, since only a weak correlation was observed. However, extreme group validity and discriminative validity were present. The SQUASH resulted in higher physical activity estimates on average than the ActiHeart, and those with a discrepancy in total physical activity measured by the SQUASH versus the ActiHeart were younger and more often men than the reference group.

### 4.1 Amount of physical activity

We found higher physical activity levels according to SQUASH than according to ActiHeart (mean difference 47 MET h/week (SD 63)). These results are in line with a small study that compared SQUASH with another accelerometer (ActiGraph) in 39 elderly participants. In this study, the mean time of physical activity per week was 1741 ​min (SD 1227) according to SQUASH and 661 ​min (SD 475) according to ActiGraph [10]. In contrast, in another small study where SQUASH was compared with doubly labelled water [30]. With this method, physical activity is estimated based on the metabolic rate to eliminate certain isotopes that are administered in a solution [31]. The study did not find a difference between SQUASH and doubly labelled water in physical activity energy expenditure (mean difference 126 ​kcal/day (95% limits of agreement: -1207 to 1459 ​kcal/day)). However, this was a small study in 17 adolescents with a very different “objective” physical activity instrument than our study, and might therefore not be comparable with our study.

The difference between the SQUASH and the ActiHeart we found might be explained by different underlying methods of physical activity measurement. Self-reported instruments such as the SQUASH are prone to response biases such as recall bias and social desirability bias, which might lead to participants overestimating their activity level [8]. Furthermore, the SQUASH is used retrospectively to investigate a week in last month, while ActiHeart was administered prospectively for four days. Consequently, these did not measure the same period which could lead to differences in outcome between these instruments. Furthermore, this relatively short wear time of ActiHeart might make it not fully representative of habitual physical activity, and increase the risk of a “Hawthorne effect” (changing physical activity behaviour due to being monitored by ActiHeart) [32].

### 4.2 Construct validity

The weak convergent validity of the SQUASH we found (rho ​= ​0.20) was in accordance with previous conducted studies. A Dutch study reported correlations separately for “light intensity” and for “moderate to high intensity” physical activity [15], which ranged widely from 0.08 for moderate/high intensity activity in men of Turkish descent (n ​= ​43) to 0.41 in women of Dutch descent (n ​= ​44). Correlation for most groups was weak. A study compared the SQUASH with an ActiGraph accelerometer, which was worn for one week in 115 Dutch ankylosing spondylitis patients [14]. The correlation between total physical activity expenditure of the SQUASH and the ActiGraph was 0.35. A third study investigated 37 Dutch participants (18–65 years, 70% men) and used as a reference a Computer Science and Applications Inc. (“CSA”) accelerometer for two weeks [9]. Correlation between this accelerometer and SQUASH total physical activity was 0.45 (“moderate”). This higher correlation than our finding might be explained by a longer wear time (two weeks instead of four days in our study), or with the different way this accelerometer assesses physical activity, because it does not rely on heart rate for activity estimation like the ActiHeart, but solely on accelerometery. Extreme group validity of the SQUASH was present in our study. This is in line with a Japanese study [13], which demonstrated lower SQUASH outcomes for participants with hip or knee osteoarthritis versus those without hip or knee osteoarthritis. Concerning discriminative validity, we did not find any previous studies investigating the SQUASH.

Given that we did not find convergent validity of the SQUASH, but did find discriminative and extreme group validity, our study suggests that construct validity of the SQUASH is sub-optimal. However, convergent validity in our study might be underestimated due to two reasons; the SQUASH and the ActiHeart did not measure the same follow-up period, and the short wear time of the ActiHeart of only four days might not be fully representative of habitual physical activity [32]. Also, weekend days are underrepresented as participants received the ActiHeart on a weekday. Therefore, further validation of the SQUASH is desirable. The feasibility of the SQUASH is confirmed by the negligible number of participants who did not fully complete the questionnaire (121/6.671, 2%). Consequently, if convergent validity of the SQUASH could be demonstrated, the SQUASH might be the option of choice for investigating physical activity. It is especially a good option in specific study settings, for example when long-term objective measurement is deemed too burdensome, or if shortness of the questionnaire is deemed important in physical activity questionnaire choice.

### 4.3 Participant characteristics associated with difference between SQUASH and ActiHeart

We found male participants and younger participants (among the middle-aged) to have a higher likelihood for a difference between SQUASH and ActiHeart outcome than female and relatively elderly participants, both with regards to overestimating the SQUASH compared with the ActiHeart, as well as underestimating the SQUASH compared with the ActiHeart. For several other participant characteristics, we found a difference between the reference quintile and other quintiles of SQUASH minus ActiHeart activity. However, due to a lack of “dose-response” relationship between the quintiles for these characteristics, the true underlying role of these characteristics in the discrepancy between SQUASH and ActiHeart remains unclear.

The relatively high number of men we found with self-reported activity higher than objectively measured outcome is in line with a study that compared the International Physical Activity Questionnaire (IPAQ) with the ActiGraph accelerometer in 1751 participants from the Norwegian general population (mean age 48, 50% men) [33]. The study found higher physical activity reported by the IPAQ for men than for women (367 versus 293 ​min per day of being active), while time per day of physical activity according to ActiGraph was comparable between men and women (344 ​min versus 347). We did not find any previous literature on the topics of the relatively high number of males we found with self-reported physical activity outcome lower than the objective outcome, as well as for the relatively younger participants having a higher risk of a difference between questionnaire and accelerometer-reported physical activity compared with older participants.

### 4.4 Strengths and limitations of this study

Our study has several strengths. A key strength is the large study population. Other strengths are the wide range of outcomes of our study; we are the first, to our knowledge, to investigate convergent, extreme group, and discriminative validity of the SQUASH. Notably, there are some limitations to our study. As mentioned earlier, the SQUASH and the ActiHeart did not measure the same follow-up period, and the short wear time of the ActiHeart might not be fully representative of habitual physical activity. Another limitation to our study is a possible lack of generalizability of our physical activity outcomes of the SQUASH to individuals from other countries. As the SQUASH was developed in the Netherlands, it might apply specifically to Dutch activity patterns. For example, two of the questions in the SQUASH concern cycling, which is often done for commuting in The Netherlands but less so in most other countries (Cavill, 2006) [34]. Therefore, further validation of the SQUASH using study populations from different countries is recommended.

In conclusion, we demonstrated discriminative and extreme group validity of the SQUASH. However, convergent validity was not found due to a low correlation with the ActiHeart. The construct validity of the SQUASH therefore seems sub optimal. Physical activity reported by SQUASH was generally higher than reported by ActiHeart, and male participants and relatively young participants appeared more at risk for a difference between these two instruments than female and relatively elderly participants. Whether the differences between SQUASH and ActiHeart are e.g. due to differences in methodology, different underlying domains, limitations to our study, or reflect true differences needs further investigation in different study populations. Also, we recommend to identify opportunities to improve the SQUASH, as well as further validation of other physical activity instruments. Within a study, we recommend to use the same physical activity instrument for all participants.

## Author contributions

Each author contributed to design of the study, interpretation of the data and critically revising the article. All authors gave final approval of the submitted article.

## Funding support

The NEO study is supported by the participating Departments, the Division and the Board of Directors of the Leiden University Medical Center, and by the Leiden University, Research Profile Area ‘Vascular and Regenerative Medicine’. This work was supported by the Dutch Arthritis Foundation LLP-24. MK has received financial support from the Dutch Arthritis Foundation.

## Declaration of Generative AI and AI-assisted technologies in the writing process

No generative AI or AI-assisted technologies were used for any aspect of this manuscript.

## Conflict of Interest

All other authors have declared no conflicts of interest.
