# Supplementary material for: Validation of the SQUASH physical activity questionnaire using accelerometry: The NEO study
Source: Osteoarthr Cartil Open. 2024 Mar 18;6(2):100462. doi: 10.1016/j.ocarto.2024.100462 (PMC10992721; doi:10.1016/j.ocarto.2024.100462)
Supplement: Multimedia component 1 [file mmc1.pdf]

SQUASH versus ActiHeart-measured physical activity in the middle-aged general population: the NEO study – supplement 1: amount of missing data

	Number of missing cases (% of all participants)
**General participant characteristics**	
Age	0 (0%)
Sex	0 (0%)
BMI	0 (0%)
Ethnicity	14 (0%)
Education	15 (0%)
Number of comorbidities	58 (1%)
Knee OA^	15 (0%)
Hand OA^	11 (0%)
SF-36 PCS	70 (1%)
SF-36 MCS	70 (1%)
**Physical activity outcomes**	
SQUASH total activity	0 (0%)
Light activity	0 (0%)
Moderate activity	0 (0%)
Vigorous activity	0 (0%)
ActiHeart activity (MET h/week) (distributed to random subset of 14% of participants)	9 (1% of the random subset)
SQUASH minus ActiHeart activity (MET h/week)	9 (1% of the random subset)

Results are based on analyses weighted towards the BMI distribution of the general population. Numbers represent number (percentage). Abbreviations: SQUASH=Short Questionnaire to Assess Health-Enhancing physical activity. BMI=body mass index (kg/m^2), OA=osteoarthritis, h = hour, SF=short form, PCS=Physical Component Scale, MCS=Mental Component Scale, MET=Metabolic Equivalent of Task. ^=Osteoarthritis according to the clinical criteria of the American College of Rheumatologists.
