# Supplementary material for: Validation of the SQUASH physical activity questionnaire using accelerometry: The NEO study
Source: Osteoarthr Cartil Open. 2024 Mar 18;6(2):100462. doi: 10.1016/j.ocarto.2024.100462 (PMC10992721; doi:10.1016/j.ocarto.2024.100462)
Supplement: Multimedia component 2 [file mmc2.pdf]

SQUASH versus ActiHeart-measured physical activity in the middle-aged general population: the NEO study – supplement 2: all analyses of table 2 adjusted for age and sex

Table 2. Participant characteristics of each quintile of participants concerning SQUASH minus ActiHeart physical activity, and differences with the reference quintile in these characteristics. Q1 is the lowest quintile of SQUASH minus ActiHeart activity and Q5 the highest.

Total group with ActiHeart	**Q1**	**Q2** (reference quintile)	**Q3**	**Q4**	**Q5**
SQUASH minus ActiHeart (MET h/week, range)	-161;-6	-6;27	27;61	61;91	91;411
Age	56.5 (6.2)	57.4 (6.0)	55.4 (6.1)	55.7 (5.7)	55.3 (6.0)
Difference (adjusted only for sex)*	*-1.1 (-2.4;0.2)*	-	*-2.0 (-3.2;-0.7)*	*-2.0 (-3.2;-0.7)*	*-2.5 (-3.8;-1.2)*
Sex (male) §	89 (50%)	54 (30%)	61 (33%)	78 (44%)	91 (50%)
OR† (adjusted only for age)	*2.4 (1.5;3.7)*	-	*1.2 (0.8;1.9)*	*2.0 (1.3;3.2)*	*2.8 (1.8;4.5)*
BMI	26.8 (3.8)	26.0 (4.0)	26.4 (4.5)	25.7 (4.5)	26.3 (4.6)
*Difference**	*0.5 (-0.4;1.3)*	-	*0.5 (-0.4;1.4)*	*-0.5 (-1.5;0.4)*	*0.2 (-0.8;1.1)*
Comorbidity§	46 (27%)	52 (30%)	30 (17%)	50 (29%)	35 (20%)
OR†	*0.9 (0.6;1.5)*	-	*0.5 (0.3;0.9)*	*1.1 (0.7;1.7)*	*0.6 (0.4;1.0)*
Knee OA^, yes§	38 (21%)	28 (15%)	14 (8%)	24 (13%)	25 (13%)
OR†	*1.8 (1.0;3.2)*	-	*0.5 (0.3;1.1)*	*1.0 (0.6;1.9)*	*1.1 (0.6;2.1)*
Hand OA^, yes§	11 (5%)	27 (15%)	13 (8%)	27 (15%)	29 (16%)
OR†	*0.3 (0.2;0.9)*	-	*0.5 (0.2;1.0)*	*1.4 (0.8;2.6)*	*1.4 (0.7;2.5)*
SF-36 PCS	54 (8)	53 (10)	54 (7)	54 (8)	55 (8)
*Difference**	*1 (-1;3)*	-	*1 (-1;2)*	*1 (-1;3)*	*2 (0;3)*

Results are based on analyses weighted towards the BMI distribution of the general population. Numbers represent mean (standard deviation) unless specified otherwise. SF-36 scores are norm based with mean of 50, higher scores are better. Abbreviations: h = hour, MET=Metabolic Equivalent of Task, OR = odds ratio, CI = confidence interval, SQUASH=Short Questionnaire to Assess Health-Enhancing physical activity. BMI=body mass index, OA=osteoarthritis, SF=short form, PCS=Physical Component Scale, ^=Osteoarthritis according to the clinical criteria of the American College of Rheumatologists. *=mean difference compared with the reference quintile (quintile 2), with 95% confidence interval, § = number (percentage), †=odds ratio for the concerning outcome compared with the reference quintile, with 95% confidence interval,.
